# A Bayesian Record Linkage Approach that Adjusts for Variables in One File

**DOI:** 10.23889/ijpds.v9i5.2552

**Published:** 2024-09-18

**Authors:** Gauri Kamat, Mingyang Shan, Roee Gutman

**Affiliations:** 1 Brown University; 2 Eli Lilly and Company

In many healthcare and social science applications, information about units is dispersed across multiple data files. Linking records across files is necessary to estimate associations between variables exclusive to each of the files. Common record linkage algorithms only rely on similarities between linking variables common to all the files. Moreover, analysis of linked files often ignores errors that may arise from incorrect or missed links. Bayesian record linking methods allow for natural propagation of linkage errors, by jointly sampling the linkage structure and the model parameters. We extend an existing Bayesian record linkage approach to integrate associations between variables exclusive to each file being linked. We show analytically, and using simulations, that the proposed method improves the linkage process, and results in accurate statistical inferences. We apply the proposed method to link Meals on Wheels (MOW) recipients to Medicare Enrollment records, and examine the relationship between activities of daily living and healthcare utilization among MOW recipients.

